# Resprouting grasses are associated with less frequent fire than seeders

**DOI:** 10.1111/nph.17069

**Published:** 2020-12-06

**Authors:** Kimberley J. Simpson, Emma C. Jardine, Sally Archibald, Elisabeth J. Forrestel, Caroline E. R. Lehmann, Gavin H. Thomas, Colin P. Osborne

**Affiliations:** ^1^ Department of Animal and Plant Sciences University of Sheffield Sheffield S10 2TN UK; ^2^ Centre for African Ecology School of Animal, Plant and Environmental Sciences University of the Witwatersrand Private Bag 3 Witwatersrand 2050 South Africa; ^3^ Department of Viticulture and Enology University of California Davis CA 95616‐5270 USA; ^4^ School of GeoSciences University of Edinburgh Edinburgh EH8 9XP UK; ^5^ Tropical Diversity Royal Botanic Garden Edinburgh Edinburgh EH3 5NZ UK

**Keywords:** bud position, drought, fire frequency, fire intensity, life history, Poaceae, photosynthetic pathway, traits

## Abstract

- Plant populations persist under recurrent fire via resprouting from surviving tissues (resprouters) or seedling recruitment (seeders). Woody species are inherently slow maturing, meaning that seeders are confined to infrequent fire regimes. However, for grasses, which mature faster, the relationships between persistence strategy and fire regime remain unknown.
- Globally, we analysed associations between fire regimes experienced by hundreds of grass species and their persistence strategy, within a phylogenetic context. We also tested whether persistence strategies are associated with morphological and physiological traits.
- Resprouters were associated with less frequent fire than seeders. Whilst modal fire frequencies were similar (fire return interval of 4–6 yr), seeders were restricted to regions with more frequent fire than resprouters, suggesting that greater competition with long‐lived resprouters restricts seeder recruitment and survival when fire is rare. Resprouting was associated with lower leaf N, higher C:N ratios and the presence of belowground buds, but was unrelated to photosynthetic pathway.
- Differences between the life histories of grasses and woody species led to a contrasting prevalence of seeders and resprouters in relation to fire frequency. Rapid sexual maturation in grasses means that seeder distributions, relative to fire regime, are determined by competitive ability and recruitment, rather than time to reproductive maturity.

## Abstract 2

See also the Commentary on this article by Day, **230**: 406‐407.

## Introduction

Plant species persist in fire‐prone environments via two broad strategies: resprouting from surviving tissue or recruiting from seedbanks (Bond & Midgley, 2001; Pausas *et al*., 2004). The relative benefit of these two strategies (‘resprouter’ vs ‘seeder’) varies with fire regime, resulting in patterns of persistence strategies along gradients of fire frequency and intensity, as seen in several woody fire‐prone taxa (Keeley, 1986; Bellingham & Sparrow, 2000; Pausas, 2001; Knox & Morrison, 2005; Clarke & Dorji, 2008; Vilà‐Cabrera *et al*., 2008; Pausas & Keeley, 2014). However, for grasses, a globally important plant family whose distribution and success are closely linked to fire (Bond *et al*., 2005; Keeley & Rundel, 2005; Scheiter *et al*., 2012; Linder *et al*., 2018), the relationship between persistence strategy and fire regime has not been explored.

Fire regimes vary spatially and temporally (Gill, 1975; Belcher *et al*., 2010; Archibald *et al*., 2013; Keeley & Syphard, 2016) with fire frequency and intensity two fire regime characteristics relevant to understanding strategies of plant growth and persistence. Fire frequency limits potential periods of plant growth, and therefore resources available to put into organs that may aid resprouting, such as rhizomes. Intensity relates to temperatures experienced, and the potential impact on plant mortality and reproductive success (Moreno & Oechel, 1991; Wade, 1993; McCaw *et al*., 1997). Fire intensity and frequency are interdependent, as long fire return periods enable the development of significant fuel loads, potentially resulting in high‐intensity fires (Bond & van Wilgen, 1996; Archibald *et al*., 2013). Hence, plant persistence strategies tend to sort along gradients of frequency and intensity (Box 1 panels a and b; Keeley, 1986; Bellingham & Sparrow, 2000; Knox & Morrison, 2005; Pausas & Keeley, 2014). These patterns have been explained by life history theory via the principle of resource allocation (Keeley & Keeley, 1977; Bond & Midgley, 2001), which posits that plants balance the allocation of limited resources among growth, maintenance and reproduction (Silvertown & Charlesworth, 2001). Seeders maximise fitness by allocating relatively more resources than resprouters to fast growth and early reproduction to increase the likelihood of reaching reproductive maturity before the next fire. By contrast, resprouters maximise fitness by allocating relatively more resources to nonstructural carbohydrate stores and protective structures that will increase the likelihood of survival post fire (Bellingham & Sparrow, 2000).

Fire has played a major role in the historical and contemporary success of the grasses (Keeley & Rundel, 2005; Scheiter *et al*., 2012; Linder *et al*., 2018). Large areas of the tropics and subtropics are maintained as grasslands and savannas by fire, despite the climatic potential to support forest (Bond *et al*., 2005; Beckage *et al*., 2011). Grasses, in turn, fuel the most frequent fire regimes and the majority of fires on Earth (Mouillot & Field, 2005). There is growing interest in how fire influences and is influenced by grasses (Ripley *et al*., 2015; Simpson *et al*., 2016; Wragg *et al*., 2018; Russell *et al*., 2019; Simpson *et al*., 2019). Grasses persist through fire by germinating from soil‐stored seed banks or by resprouting from buds (Bond & van Wilgen, 1996). However, how resprouter and seeder grasses sort along gradients of fire frequency and intensity remains untested. Yet, it is important in understanding the consequences of stark differences in life histories that likely underpin relationships between fire regimes and persistence strategies (Pausas, 2001; Box 1 panels c and d).

The plant traits associated with seeder and resprouter strategies of fire‐prone woody species are well studied (Paula & Pausas, 2006; Vivian & Cary, 2012), but remain poorly understood in grasses. The C_4_ photosynthetic pathway, which has evolved multiple times independently in grasses (Grass Phylogeny Working Group II, 2012) and is highly efficient in warm, high‐light environments, such as fire‐prone grasslands (Tix & Charvat, 2005; Ratnam *et al*., 2011), may be important to grass resprouting ability (see Table 1 for specific predictions). A recent study by Moore *et al*. (2019) found that post‐fire survival across 52 perennial grasses was associated with C_4_ photosynthesis. Likewise, the positioning of resprouting buds is also expected to be crucial (Pausas & Paula, 2020). Buds below the soil surface (rhizomes) are likely better protected from heat than those at the soil surface (crown resprouters or species with stolons; Table 1). Leaf traits probably also differ between seeders and resprouters, as found in woody fire‐prone species (e.g. Paula & Pausas, 2006). Leaf traits associated with potential growth rates and resource investment, such as specific leaf area (SLA; Forrestel *et al*., 2014), and nitrogen (N)‐use efficiency (associated with low leaf N content), may be important in fire‐prone, N‐poor environments (Table 1; Knapp & Medina, 1999; Long, 1999; Sage, 2004; Keeley & Rundel, 2005). Likewise, leaf traits that enhance flammability, such as a high leaf carbon (C) : N ratio, occur in resprouting, shade‐intolerant grasses which require frequent defoliation (Table 1; Everson *et al*., 1988). Life history is likely to be closely linked to resprouting ability, with perennials presumably able to resprout but annuals not (Table 1). Whether this is strictly true, or if there are exceptions to this trend, is unclear.

**Table 1:** Plant traits and their predicted associations with resprouting ability in fire‐prone grasses.

Trait	Relationship with resprouting ability
Photosynthetic pathway	Resprouters are more likely to be C_4_ than C_3_ (Moore *et al*., 2019). C_4_ species are highly efficient in fire‐prone environments and may therefore have greater stored resources to resprout (Tix & Charvat, 2005; Ratnam *et al*., 2011)
Bud position	Resprouters are more likely to have buds below the soil surface (rhizome resprouters) where they are protected from intense heat (Pausas & Paula, 2020)
Specific leaf area (SLA)	Resprouters will have lower SLA than seeders (Forrestel *et al*., 2014). High SLA will aid the rapid growth of seeder species
Leaf nitrogen (N) content	Resprouters will have lower leaf N contents than seeders. Resprouters may experience fire multiple times in their lifetime and thus low‐N availability (due to N volatilisation during fire; Reich *et al*., 2001; Hernández & Hobbie, 2008). In these conditions, a high N‐use efficiency (low leaf N content) may be advantageous (Wedin & Tilman, 1990; Reich *et al*., 2001)
Leaf C : N ratio	Resprouters will have higher leaf C : N ratios than seeders. High leaf C : N ratio, which is linked to low decomposition rates and the accumulation of a highly flammable fuel load (Aerts, 1997), may be advantageous to shade‐intolerant resprouting species in maintaining an open canopy (by aiding the removal of standing dead and woody biomass; Everson *et al*., 1988)
Life history	Resprouters are more likely to be perennial than seeders. Perennial‐grass species have buds from which to regrow, which annual species may lack

Here, we explore the global relationship between fire characteristics (frequency and intensity) and the persistence strategies of fire‐prone grass species. We predict that patterns of resprouting and seeding with fire characteristics in grasses will differ from those in woody species in the ways outlined in Box 1. Based on *a priori* expectations (Table 1), we also investigate how plant traits (photosynthetic pathway, life history, bud position, SLA, leaf N content and C:N ratio) relate to resprouting ability.

Box 1 The predicted relationships between persistence strategy and fire characteristics for fire‐prone woody taxa (a and b; solid line in (a) from Bellingham & Sparrow, 2000) and grass taxa (c and d). Solid lines represent the distribution of resprouter species and dashed lines represent seeder species. Fire characteristics are frequency and intensity, and the coloration of plot backgrounds represents typical differences in characteristics between fire regimes fuelled by woody or grassy vegetation (with grass‐fuelled fires being more frequent and less intense on average; Archibald *et al*., 2013).

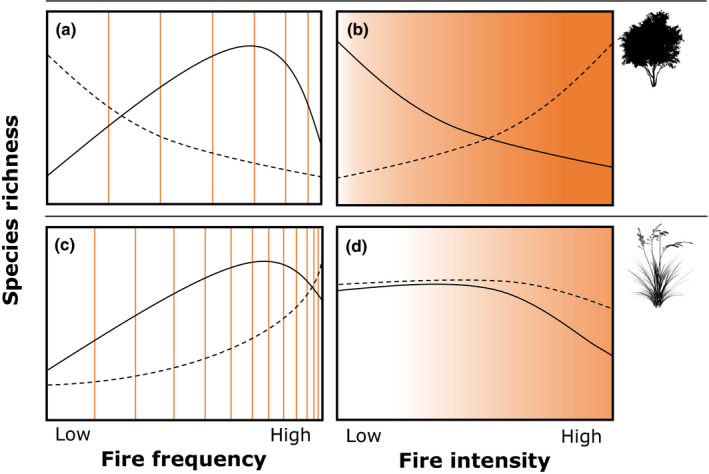

**Relationships for woody species**

Fire frequency (a) – Woody seeders (dashed lines) are susceptible to ‘immaturity risk’ (Zedler, 1995), where intervals between fires are shorter than the time taken to reach maturity (e.g. Pausas, 2001). Therefore, the seeder strategy becomes more viable as the interfire period increases and fire frequency decreases. Only seeders with rapid growth and early maturation can exist in very high fire frequencies. By contrast, the cost of storage organs to growth in low fire frequencies means that resprouting (solid lines) is more viable with increasing fire frequency (Bellingham & Sparrow, 2000). However, very high frequencies make maintaining storage organs energetically unfeasible (Vilà & Terradas, 1995; Grady & Hoffman, 2012; Fairman *et al*., 2019; Day *et al*., 2020).

Fire intensity (b) – Woody seeders are predicted to be less susceptible to high‐intensity fires than resprouters, as the seeds of many fire‐prone seeder species are protected from the heat of the fire through insulating seed‐storage structures (e.g. serotinous cones from which seeds are released following a fire) or a layer of soil, and are thus much less vulnerable to heat‐related damage than adult resprouting plants (Keeley & Fotheringham, 2000; Pausas & Keeley, 2014; Day *et al*., 2020). The buds of resprouters may be protected to an extent by insulating bark (for epicormic buds) or soil (for basal buds).

**Relationships for grass species**

Fire frequency (c) – At low fire frequencies, grass resprouters decline because of the energetic costs of maintaining buds when fire is rare. In addition, for clump‐forming resprouting species (‘crown resprouters’), the accumulation of detritus due to infrequent fire can lead to individuals becoming moribund (Knapp & Seastedt, 1986), which may be fatal (Zimmerman *et al*., 2010). At very high fire frequencies, resprouting may decline, as maintaining storage organs becomes less feasible, but will still be common (as resprouting grasses fuel the most frequent fire regimes on Earth; Mouillot & Field, 2005). In contrast to woody seeders, we expect grass seeders to be largely restricted to high fire frequencies when fire return intervals are short. Grasses generally have much shorter generation times than woody species, for example, members of the fire‐prone grass lineage, Andropogoneae, flower within months of germination (Estep *et al*., 2014). Therefore ‘immaturity risk’ is not likely to limit the distribution of grass seeders, even when accounting for the grass‐fuelled high fire frequencies. In less frequent fire regimes, we expect seeders will be outcompeted by established resprouters (Zimmerman *et al*., 2008).

Fire intensity (d) – Grass‐fuelled fires tend to be low intensity although there are exceptions (e.g. Spinifex (*Triodia* spp. dominated) grasslands of Australia experience very hot fires; Archibald *et al*., 2013). Therefore, intensity may not be a relevant fire characteristic in determining the distributions of grass seeders and resprouters, as has been found for other grass traits (Trollope *et al*., 2002; Uys *et al*., 2004; Peláez *et al*., 2013) because it does not represent a strong selection pressure. High fire intensities may cause mortality, particularly in resprouters.

## Materials and Methods

### Grass species occurrences

We extracted all georeferenced and dated occurrence records (*c*. 18.6 M records) for Poaceae taxa from the Global Biodiversity Information Facility (GBIF) web portal (GBIF.org (5 November 2019) GBIF Occurrence Download doi: 10.15468/dl.rckugp) via the R statistical computing package rgbif (Chamberlain *et al*., 2020). This analysis and all statistical analyses were carried out in the R environment (R Core Team, 2019).

Steps were taken to control the quality and suitability of occurrence records (Supporting Information Table S1 gives the number of records remaining after each cleaning step). Species names were standardised against the Kew Grass Synonymy database (Clayton *et al*., 2006) using the software package Taxonome (Kluyver & Osborne, 2013), and records were discarded if an accepted name could not be given. Longitude and latitude data were checked to ensure values were sensible using the coordinatecleaner package (Zizka *et al*., 2019; for example removal of invalid and nonterrestrial coordinates, duplicated records and records within biodiversity institutions) and were accurate to at least three decimal places, to represent the small scale over which fire regimes change. Finally, to ensure that records represented individuals from environments subject to ecologically relevant fire regimes, domesticated grass species were excluded (Kluyver, 2013) and all other species’ records were checked against Kew GrassBase distributions (Clayton *et al*., 2006). Records from highly transformed landscapes, where fire regimes are altered through changes in ignition frequency and fuel properties, were also removed. To do this, records from protected areas were identified (using the World Protected Areas map; http://www.wdpa.org/) and kept. Otherwise, a human influence index (HII) value was obtained for each record (Sanderson *et al*., 2002), which indicates how impacted was the landscape by human activities (based on population density, access, land‐use and infrastructure). Records with an HII value > 30 are from highly transformed landscapes in terms of fire characteristics (Archibald *et al*., 2013) and were excluded. Records made before 1980 were removed (a compromise between removing records taken before satellite‐derived fire datasets began and not removing a large proportion of the total records). After these cleaning steps, species were excluded if they were sampled by < 50 unique occurrence points.

### Fire characteristics

Data from the MODIS satellite were used to characterise the fire regime to which grasses were exposed. This is a *c*. 20‐yr dataset, which covers a similar time period as the GBIF location records. Therefore, although current fire regimes might not represent the evolutionary history of fire in that location, they do represent the fire regime to which the species occurring there are exposed and able to persist under.

#### Fire frequency

Fire frequency is characterised as the median fire return interval (FRI; the time between successive fires), with high frequencies associated with short FRIs. The MODIS global monthly burnt area (MCD64A1) satellite data product was used to calculate FRI values. This provides fire data for the Earth’s surface at a 500‐m resolution. Utilising characteristic changes in surface reflectance following a fire (e.g. charcoal/ash deposits, vegetation loss), as well as active fire detections, the MODIS algorithm identifies recent burn scars and provides an approximate date of burning (Giglio *et al*., 2009).

Dates of fire occurrence at each GBIF location during the *c*. 20‐yr MODIS dataset (April 2000 to January 2020) were extracted and used to quantify all interfire intervals to which each species was exposed (after Archibald *et al*., 2010). A Weibull distribution was fitted to these interval data for each species using the survival R package (R Core Team, 2019). The first and last fires of the MODIS dataset were open tailed (i.e. the limited timespan of the dataset means that the interfire interval before the first fire and after the last fire cannot be determined) and the survival analysis allows these open‐tailed intervals to be included to maximise the information provided. Estimations of the Weibull shape and scale parameters were determined for each species using data from pixels that burnt at least once during the 20‐yr period. The shape and scale parameters were used to determine the median FRI using the equation from Moritz *et al*. (2009):

$$\text{FRI}=b{\left(\log_{e}2\right)}^{\frac{1}{c}}$$

where *b* is the scale parameter and c is the shape parameter. For species in infrequently burnt places (i.e. where the fire return period is much greater than the 20 yr of the MODIS dataset), the above FRI algorithm would converge either on an unrealistically large FRI or not converge at all. We focused on species for which fire is a major disturbance and frequent enough to act as a selective pressure over their range, and hence we defined whether species were ‘fire‐prone’ or not. To do this, the proportion of records that fell within pixels that burned, relative to the total number of records for each species, was used. This proportion was plotted against the number of species and a break‐point regression fitted to the curve (Fig. S1) using the segmented library in R (Muggeo, 2003). An estimated break‐point value was calculated (0.26) and species with a proportion burned lower than this value were excluded (i.e. species that had < 26% of their occurrence records in burnt locations were excluded from fire frequency analyses). The impact of any spatial bias in the occurrence records on the proportion burned or the values of FRI was tested by subsetting the records such that there was a maximum of three records per species in each 10 km^2^ grid cell (a grid size that represents a compromise between the finer scale fire characteristic data and not removing a large proportion of the occurrence records). Values of proportion burned and FRI were highly similar between the original and subsetted datasets (*r^2^* = 0.99 and 0.95, respectively), suggesting that there is no pervasive spatial bias in the original dataset. Finally, any species with fire return intervals that were > 100 or < 1 yr were excluded. The limitations of a 20‐yr dataset mean that the results of the survival analysis were not able to resolve these fire regimes sufficiently.

The stringent data cleaning process and focus on frequently burned species resulted in a dataset containing 734 fire‐prone species (samples per species range = 53–25319; median = 270), whose distribution covers the majority of fire‐prone areas, where grasses are an important vegetation component (see Fig. S2 for the distribution of occurrence data).

#### Fire intensity

Values of fire radiative power (FRP), or the rate of radiant energy released, can be obtained from satellite measurements of middle infrared emission over actively burning areas. FRP is frequently used as a proxy for fire intensity (Dwyer *et al*., 2000; Archibald *et al*., 2013), and is available globally at 0.5° resolution from the MODIS global monthly fire location product (MCD14ML).

FRP values, measured in megawatts per 1‐km pixel, were extracted for all active fire points over the duration of the dataset (2002–2019) for each record. Any FRP values with a detection confidence < 50% were discarded. Data were grouped by species, and the 95^th^ quantile extracted. There is typically a bias towards low FRP values, due to the high variation in this measurement over the duration of a fire (Dwyer *et al*., 2000) and low values during the night. The 95^th^ quantile was therefore used, as done elsewhere (Archibald *et al*., 2013), to indicate the maximum value that a headfire could attain in a particular environment and to avoid errors caused by outliers. The 95^th^ quantile related significantly (*P* < 0.001) with mean (*r^2^* = 0.90) and median (*r^2^* = 0.75) values across all species.

### Precipitation and drought

A difficulty in determining the role of fire in driving spatial patterns of plant traits is that fire characteristics are often correlated with climate. High fire frequencies typically occur in areas with high rainfall and long dry seasons, where greater and more spatially connected fuel loads are produced (Pausas & Bradstock, 2007; Pekin *et al*., 2011). Moreover, droughts influence the success of different grass persistence strategies: both directly, through causing death of perennial grasses and recruitment opportunities for seeder grasses (O'Connor, 1995), and indirectly, through changing the amount and the moisture content of fuels, and thus fire regimes. Drought is therefore expected to be a strong environmental filter (O'Connor, 1995). We used Foley’s drought index (FDI; Foley, 1957; Fensham *et al*., 2009) here to account for the influence of drought on grass persistence strategy that is independent of fire characteristics. The FDI takes the deviations of monthly measurements (i.e. actual rainfall for a period minus the expected rainfall for the same period) from long‐term monthly averages and normalises it with respect to average annual rainfall (to allow for comparison across rainfall zones). It is therefore a measure of drought intensity that is not directly correlated with rainfall. To investigate drought at global scales, monthly rainfall values at 0.5 degree scale were used following Lehmann *et al* (2014). For each grid cell, the FDI was calculated for each month of each yr as actual annual rainfall for 3 yr prior less the expected (long‐term average) rainfall for that period, divided by the mean annual precipitation for the period 1901–2003. Monthly values for each location record were averaged per species.

### Plant traits

Grass species’ persistence strategies through fire were collected from a literature review, drawing upon large plant fire‐response databases (e.g. Cook *et al*., 2005; Crowley *et al*., 2007; Paula *et al*., 2009), journal papers (e.g. du Toit *et al*., 2014; Marais *et al*., 2014; Moore *et al*., 2019) and the grey literature (such as Government reports regarding fire management strategies or control of invasive species). Data from sources already known to the authors were extracted first, followed by a targeted approach for species for which we had fire characteristic data. The latter was achieved by using the Web of Knowledge and Google Scholar search engines (to incorporate both the primary and grey literature) for the term ‘[species name] fire response’. We classed species as either ‘seeders’ or ‘resprouters’ based on the response specifically to fire and not to other disturbances, as fire not only defoliates a plant (similar to other disturbances), but it also has lethal effects on meristem tissues (Pausas *et al*., 2016). Resprouters were defined in a number of ways depending upon the type of data available. For categorical data, resprouting species were defined as those where resprouting is the main post‐fire persistence strategy. In a few cases, the ability to resprout was given in relation to fire characteristics (e.g. ‘plants will recover from an occasional, but not annual, fire’), and these were classed as resprouters because they showed an ability to resprout after fire. For quantitative data, species that experienced < 30% mortality when subjected to 100% leaf scorch were classed as resprouters (*sensu* Crowley *et al*., 2007). Conversely, a species was classified as a seeder if the main post‐fire persistence strategy was germinating from seed (which could be either from a stored soil seedbank or from seeds dispersed into a recently burnt area), or if plants of this species experienced > 70% mortality when subjected to 100% leaf scorch. In addition, annual species for which no response to fire could be found by searching the literature were assumed to be seeders, unless there was evidence to the contrary (*n* = 27). The seeder/resprouter dichotomy represents the standard way in which a species recovers from fire, but we acknowledge that these strategies are not mutually exclusive, with a number of species able to do both (i.e. facultative seeders; Pausas & Keeley, 2004). The resulting dataset, consisting of 763 taxa, of which 64% of species are classed as resprouters (a similar proportion found in other plant taxa and communities; Clarke, 2002 (71%); Clarke & Dorji, 2008 (60%)), is available via Dryad entry doi: 10.5061/dryad.3bk3j9khn.

Data on plant traits which we thought may be associated with the ability to resprout (Table 1) were collected from a number of sources. Photosynthetic type was acquired from Osborne *et al*. (2014), and bud position from the Kew GrassBase dataset (Clayton *et al*., 2006). The latter involved the assignment of categories of bud position: belowground buds (species with rhizomes) or buds at ground‐level (species with stolons or crown resprouters, defined as caespitose species with neither rhizomes nor stolons). Species‐level leaf traits (specific leaf area (SLA), foliar N content and foliar C : N ratio) were obtained from a dataset of 279 grass species (Jardine *et al*., 2020). A principal components analysis (using the ‘*princomp*’ function; R Core Team, 2019) collapsed the variance in these three leaf traits into two axes that together accounted for 98.7% of total variation (69.1% on dimension 1; 29.6% on dimension 2; Fig. S3). Foliar N content and C : N ratio loaded most heavily on dimension 1, with N content being positively correlated (*r^2^* = 0.88; *P* < 0.001) and C/N ratio being negatively correlated (*r^2^* = 0.82, *P* < 0.001) with this dimension. SLA loaded heavily in dimension 2, and was positively correlated with this dimension (*r^2^* = 0.69, *P* < 0.001; Fig. S3).

#### Phylogeny

To account for evolutionary relationships among grass species, we used a completely sampled, dated Bayesian phylogeny of grasses that incorporates 11 297 grass taxa (Forrestel, 2015) and combined molecular and taxonomic data following the methods of Jetz *et al*. (2012) and Thomas *et al*. (2013). A maximum clade credibility tree was inferred from the resulting distribution of trees using MrBayes (Ronquist *et al*., 2012), and subset to include species in our study (available via Dryad entry doi: 10.5061/dryad.3bk3j9khn).

### Data analysis

The relationships between grass persistence strategy and fire characteristics (frequency and intensity) were analysed using phylogenetic logistic regression (Ives & Garland, 2010) implemented in the phylolm package in R (Ho & Ane, 2014). The response variable was persistence strategy, coded as either 0 (seeder species) or 1 (resprouter species). Either fire frequency or fire intensity was selected as an explanatory variable. The drought index, FDI, was not highly correlated with either fire frequency (Pearson's product moment correlation coefficient = 0.05) or intensity (correlation coefficient = 0.43) and so was included as an additional explanatory variable. Any significant interaction terms between the explanatory variables were included in the final models. Fire frequency and intensity values were log‐transformed to improve normality. The ‘Logistic_MPLE’ (Maximised Penalised Likelihood) method was used, and all analyses were performed with 10 000 bootstrap replicates. Sample sizes are given in Table S2.

To determine how plant traits (photosynthetic pathway, bud position, life history, leaf trait PCA axis one and two) associate with persistence strategy, phylogenetic binary logistic regressions were fitted (as above) with persistence strategy as the response variable and each plant trait in turn selected as the explanatory variable (see Table S2 for sample sizes).

Relationships between fire frequency, fire intensity and drought were determined using phylogenetic generalised least squares with the ‘*pgls*’ function in the caper package (Orme *et al*., 2018).

In these analyses we used species‐level data. We recognise that some species show large variability in their traits (e.g. Moreira *et al*., 2012), and in the environmental conditions they can persist in, but we could not account for within‐species variability because the trait data and the location data did not coincide.

## Results

### Grass persistence strategy relationships with fire characteristics

**Fig. 1:**
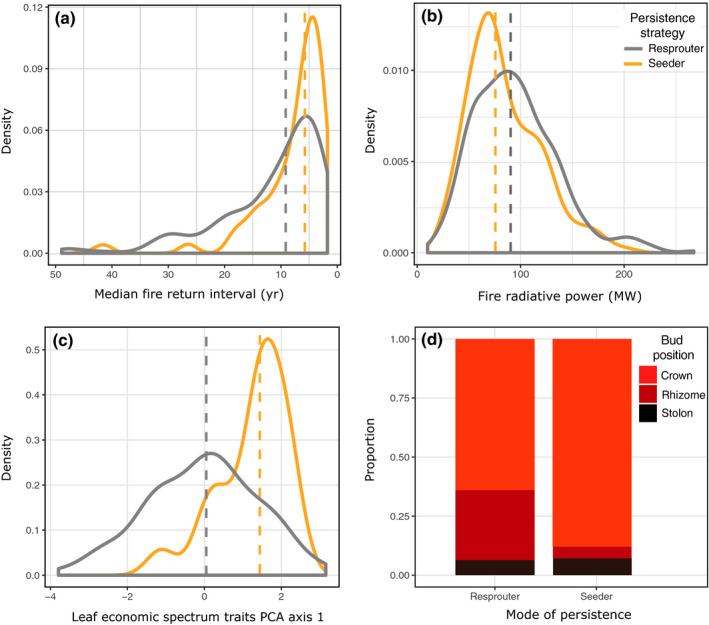
Fire characteristics and plant traits associated with persistence strategy in fire‐prone grass species. (a) Seeders are associated with more frequent fire (i.e. shorter fire return intervals), and (b) lower intensity fire than resprouters (intensity values are 95^th^ percentile of fire radiative power). (c) Resprouters have lower values for leaf trait PCA axis one than seeders (i.e. lower values of leaf N content and higher leaf C : N ratio). (d) A higher proportion of resprouters have buds positioned belowground (in the form of rhizomes) than seeders. Dashed lines in density plots represent median values for either resprouters (grey) or seeders (orange). Sample sizes: (a) 332 species; (b) 550 species; (c) 114 species; (d) 561 species. The *x*‐axis in (a) is reversed (so that high fire frequencies are to the right) and constrained to a maximum of 50 yr for ease of viewing (which accounts for 97% of data).

**Fig. 2:**
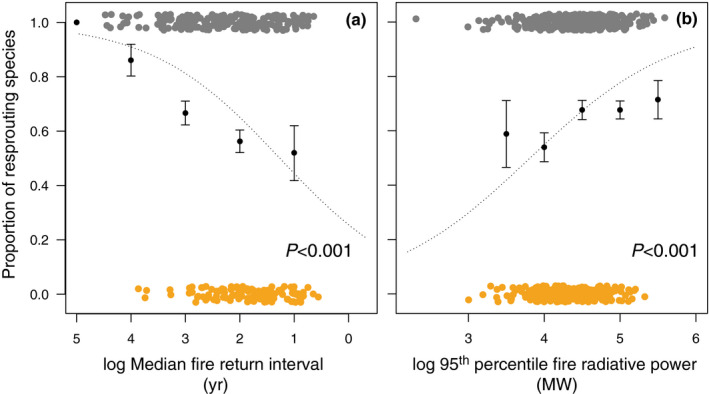
The proportion of resprouting grass species increases as fire frequency goes down (a) and fire intensity (b) goes up. The dashed line is the relationship as predicted by phylogenetic logistic regression with the effect of drought accounted for. Both fire characteristics are log‐transformed to improve normality of the data. Jittered points show the distribution of seeder (orange points) and resprouter (grey points) species. Black points are mean proportion values for data binned by fire characteristic values with each bin representing one log‐transformed unit (e.g. in (a), logged fire return intervals are divided into the following bins: 0–1, 1–2, 2–3, 3–4, 4–5). Error bars represent the standard error of the mean for the binned data. The *x*‐axis in (a) is reversed so that high fire frequencies (short fire return intervals) are to the right.

Both seeders and resprouters can persist in high fire frequencies (i.e. short FRIs; Fig. 1a; modal FRIs: 4.4 yr for seeders and 5.7 yr for resprouters). However, seeders are excluded when fire is infrequent whereas resprouters can tolerate a range of fire frequencies. This difference means that resprouters are associated with significantly longer FRIs than seeders (coefficient = 0.84, bootstrapped 95% confidence interval (CI): 0.53–1.20; *n* = 332, *P* < 0.001). The median FRIs for resprouters is 8.1 yr (standard deviation (SD): 15.5 yr) in contrast with 5.7 yr for seeders (SD: 7.5 yr; Figs 1a, 2a). This effect was over and above the significant relationship between drought and grass persistence strategy, with resprouters associated with a lower intensity multiannual drought (coefficient = 1.30 (95% CI: 0.42–2.15); *P* = 0.007) than seeders. Resprouting was the predominant persistence strategy across the range of fire frequencies (Fig. 2a). There was no significant interaction between fire frequency and drought, providing evidence that these environmental factors are independently associated with grass persistence strategy.

Grass seeders typically experience lower intensity fire than grass resprouters (coefficient = 1.05; 95% CI: 0.87–1.30, *n* = 550, *P* < 0.001; Figs 1b, 2b). The mean 95^th^ percentile of FRP was 75.1MW (SD: 35.2 MW) for seeders and 90.3 MW (SD: 40.8 MW) for resprouters. Again, there was also a significant association between drought and grass persistence strategy that was independent of fire (coefficient = 1.61 (95% CI: 0.94–2.30), *P* < 0.001).

### Fire characteristics

**Fig. 3:**
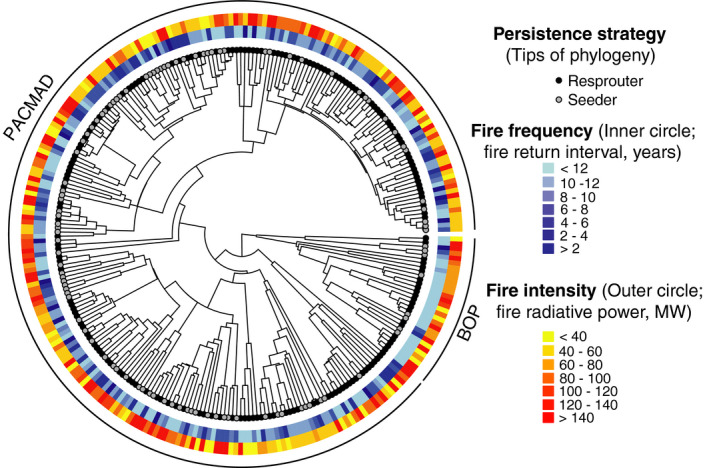
Fire characteristics and persistence strategies of fire‐prone grasses. Phylogeny tips reflect persistence strategy (resprouter/seeder). The inner circle represents fire frequency values, as measured as the median fire return interval in years (darker shades of blue are higher fire frequencies). The outer circle shows values of fire intensity, measured as fire radiative power (MW). The 278 species shown are those with data for both fire frequency and intensity, and persistence strategy. PACMAD and BOP are the two major Poaceae clades

Fire‐prone grasses experience a range of fire frequencies (FRI range: 1.0–94.1 yr; Fig. 3). The species‐level median FRI is 6.9 yr, which fits closely with the estimated global mean value for tropical grasslands and savannas (e.g. 6.5 yr for the period 1900–2000; Mouillot & Field, 2005). Species average fire intensity values ranged from 20–267 MW (median = 85 MW) per 1 km pixel (Fig. 3). These values are considered ‘low’ to ‘medium’ fire intensities (category 1 or 2 *sensu* Ichoku *et al*., 2008), which is consistent with what is expected from grass‐fuelled surface fires (Archibald *et al*., 2013).

As predicted, fire frequency and intensity are inversely correlated, such that grass species that experience more frequent fire, also experience lower intensity fire (*F*_1,491_ = 82.2, *P* < 0.001, *r^2^* = 0.14; Fig. S4). Intense droughts are associated with the occurrence of very intense fire (*F*_1,548_ = 128.5, *P* < 0.001, *r^2^* = 0.19; Table 2; Fig. S4), and there is some evidence that extreme droughts are also less likely to co‐occur with frequent fire regimes (*F*_1,330_ = 9.91, *P* = 0.002, *r^2^* = 0.03; Table 2; Fig. S4).

**Table 2:** Associations of plant persistence strategy (coded either as 0 for seeders or 1 for resprouters) with two fire characteristics, median fire return interval (a measure of frequency; a) and 95^th^ quantile of fire radiative power (a measure of intensity, b), and drought, across fire‐prone grass species (*n* = 332 in a; *n* = 550 in b).

(a)	Estimate (bootstrapped 95% CI)	Z value	*P*‐value^†^
Intercept	0.37 (−0.72 to 1.41)	0.62	0.52
Fire frequency	0.84 (0.53–1.20)	4.84	**<0.001**
Drought^§^	1.29 (0.42–2.15)	2.71	**0.006**
(b)			
Intercept	−2.30 (−2.89 to −1.79)	−2.53	**0.011**
Fire intensity	1.05 (0.87–1.30)	4.43	**<0.001**
Drought ^§^	1.61 (0.94–2.30)	3.91	**<0.001**

^†^ Significant model terms (*P* < 0.05) are in bold.

^§^ Drought is characterised by the rainfall deficit standardised by mean annual precipitation (Foley's drought index; Foley, 1957), with more negative values representing more extreme drought.

### Plant traits associated with persistence strategy

As predicted, resprouting ability is strongly related to life history (coefficient = 4.61, bootstrapped 95% CI from 4.01 to 5.28, *n* = 728, *P* < 0.001) with the majority of resprouters being perennials (97%) and the majority of seeders being annual species (76%). There are exceptions, however, with 14 annual species able to resprout after fire (e.g. *Panicum verrucosum*, *Agrostis venusta* and *Oplismenus hirtellus*) and 60 perennials persisting through fire only as seeds (e.g. *Digitaria diffusa, Paspalum conjugatum, Enneapogon lindleyanus;* full list available via Dryad entry doi: 10.5061/dryad.3bk3j9khn). The resprouter strategy was unrelated to photosynthetic pathway (coefficient = 0.21 (95% CI: −0.17 to 0.58), *n* = 597, *P* = 0.31), with 61% of C_3_ species and 63% of C_4_ species being resprouters. In terms of bud position, resprouters were more likely to have rhizomes (i.e. underground buds) than seeders (30% vs 4%, respectively; coefficient = 2.22 (95% CI: 1.41–3.19), *n* = 561, *P* < 0.001) but did not differ in the proportions of other bud positions (*P* > 0.05; Fig. 1d).

Resprouters and seeders differed in their leaf traits relating to N content and C : N ratio (PCA dimension one; coefficient = −0.62 (95% CI: −1.13 to −0.21), *n* = 114, *P* = 0.007) but not SLA (PCA dimension two; coefficient = −0.02 (95% CI: −0.54 to 0.61), *n* = 114, *P* = 0.91). Specifically, resprouters were associated with lower leaf N content and higher C : N ratio than seeders (median PCA dimension one scores: 0.04 (resprouter) vs 1.44 (seeder); Fig. 1c).

## Discussion

Grasses differ substantially from fire‐prone woody species in the sorting of persistence strategies along fire gradients, emphasising the need to develop theory and understanding about plant–fire relationships among diverse life forms that have different patterns of growth, fecundity and resource allocation (Pausas, 2001). The variable patterns between woody and grassy species persistence strategies related to fire frequency suggests diverse processes shape different fire‐prone ecosystems. Whilst ‘immaturity risk’ likely excludes woody seeder species from regions with frequent fire, this was not the case for grasses. Grass seeders are associated with higher relative fire frequencies than grassy resprouters, suggesting that, despite FRIs as short as 1 yr, sexual maturity is reached between fires. The precocious embryo of grass seed, in which root, shoot and haustorial structure are already differentiated (Gibson, 2009), has been credited with the rapid establishment and development of grasses (Linder *et al*., 2018), with individuals being able to flower in as little as 6 wk (Cope *et al*., 2009). Seeder establishment is likely limited by high resprouter densities, as the size and storage reserves of resprouters enable rapid occupation of space after fire (Keeley, 1986; Myerscough *et al*., 1995; Pausas & Keeley, 2014). Consistent with this are observations that resprouter grasses can reach considerable ages within grasslands (e.g. the lifespan of North American resprouting grasses can exceed 30 yr; Lauenroth & Adler, 2008), with seed recruitment rare (Defossé *et al*., 1997; Milton & Dean, 2000; Zimmerman *et al*., 2008). By contrast, under very high fire frequencies, resprouting may become energetically inviable, and seeders can dominate, such as in northern Australian savannas dominated by annual *Sorghum* species (Miles, 2003). Our results are consistent with the ‘gap‐dependent’ model which has been used to explain patterns of persistence strategy in some woody species (Keeley *et al*., 2016; but see Cowling *et al*., 2018). How the lifetime reproductive effort of grass seeders and resprouters differs is unclear. Data from Mediterranean shrubs suggest that seeders are smaller seeded than resprouters (Verdú, 2000), and so might produce more seeds in a limited number of reproductive events.

Grassy resprouters peaked at fire frequencies of *c*. 5 yr return intervals (Fig. 1a), in contrast with woody resprouters typically restricted to fire frequencies of 5–25 yr return intervals in shrublands and woodlands (Le Maitre & Midgley, 1992). For grass resprouters, tolerance of frequent fire is enabled by resprouting from basal meristems. Further, crown resprouters typical in productive ecosystems require regular removal of standing dead biomass to prevent them becoming moribund through self‐shading (Everson *et al*., 1988; Zimmerman *et al*., 2010) where litter build‐up can result in the death of resprouting grasses (Knapp & Seastedt, 1986). Interestingly, although perennial grasses persist at defoliation frequencies of more than 1 yr (Danckwerts & Nel, 1989; Danckwerts, 1993), we found that as in woody plants, very high fire frequencies are correlated with fewer resprouters. It is worth noting that both mammalian grazing and fire typify grassy ecosystems, and whilst we focus on fire, grazing also likely shapes the patterns reported here, with resprouters associated with heavily grazed environments (Solofondranohatra *et al*., 2020), and where heavy grazing excludes fire (Archibald & Hempson, 2016). In Africa, the intensity of mammalian grazing peaks at lower rainfall values (670 mm MAP) in contrast with the peak of fire frequency at 990 mm MAP (Archibald & Hempson, 2016). As expected, both grass seeder and resprouter species decline under low fire frequencies (although species not considered ‘fire‐prone’, because their records primarily occurred in areas unburnt for > 20 yr, were excluded from our analyses), presumably because woody‐plant life forms, correlated with lower fire frequencies, dominate landscapes. Consistent with this are abundant observations of forest encroachment into grass‐dominated areas upon fire suppression (e.g. Hoffmann *et al*., 2012).

Differences in fire intensities between grass and woody‐plant fuelled fires may explain their contrasting patterns in persistence strategies. Grass‐fuelled fires are typically lower intensity than those fuelled by woody species, with temperatures less than 200°C (cf. *c*. 400°C for fires in shrublands/forests; Bailey & Anderson, 1980), due to the low fuel loads of fine vegetation. These differences in fire intensity suggest that grass resprouters may be less vulnerable to heat‐induced mortality than woody resprouters. We found that grass resprouters were associated with higher fire intensities than seeders, the opposite pattern to woody species (e.g. Day *et al*., 2020; although many woody resprouting species can survive and thrive in high‐intensity crown fires regimes, see Pausas & Keeley, 2017 and references within). The rapid combustion of grass fuels (Simpson *et al*., 2016), and resultant low heat residence time, in combination with the cooler fire temperatures, means that resprouting grasses often survive after their aboveground biomass is scorched. Likewise, grass seeds can survive grassland fire temperatures (Gashaw & Michelsen, 2002), or disperse into recently burnt areas using anemochory, epizoochory or autochory (Ernst *et al*., 1992). Indeed, evidence relating to other grass traits suggests that fire intensity is not an especially strong selection pressure on grasses (Trollope *et al*., 2002; Uys *et al*., 2004; Peláez *et al*., 2013). The association between resprouting and high‐intensity fire may result from the ability of resprouters to accumulate more biomass than seeders between fires (e.g. fuel loads in perennial‐grass‐ vs annual‐grass‐dominated Australian savannas: *c*. 200 vs 50–100 g m^2^; Lacey *et al*., 1982), because they can produce more biomass in a period of time and also have longer intervals between fires. Resprouters may therefore be causing more intense fires rather than being better adapted to surviving them.

Drought was related to grass persistence strategies independently of fire. Studies of woody plants show that resprouters and seeders use alternative regulatory strategies with respect to water status, and are thus affected by water limitation differently (McDowell *et al*., 2008; Pausas *et al*., 2016). Resprouters tend to be ‘dehydration avoiders’, which tightly regulate their water status through drought, using strict stomatal control, deep roots and a high‐water storage capacity. By contrast, seeders tend to be ‘dehydration tolerators’, which allow their water status to greatly decline through drought because they have shallow roots, weak stomatal control and a limited ability to store water (Clarke & Knox, 2002; Meentemeyer & Moody, 2002; Pausas & Bradstock, 2007; Keeley *et al*., 2012). They do however avoid injury and are able to continue gas exchange through a water deficit (unlike drought avoiders), providing drought is not too intense. Long droughts are expected to be problematic for drought‐avoiding resprouter species, due to the carbon deficits that arise from protracted stomatal closure, resulting in respiratory demands not being met (Plaut *et al*., 2012). The association of grass resprouters with less intense droughts found here is consistent with these findings from woody species. Studies comparing the rooting depths and plant‐water physiology of annual and perennial grasses (as proxies for seeder and resprouter species; Schenk & Jackson, 2002; Vaughn *et al*., 2011) further support this differentiation of persistence strategy by drought regime. Therefore including drought helped us to explain some of the outliers in our dataset: whilst most seeders are associated with very frequent fire, some seeder species are associated with infrequent fire (20+ yr FRI; Fig. 1a) such as *Cenchrus prieurii* and *Vulpia microstachys*. These species are found in drought‐prone environments and the reseeding strategy is probably an adaptation to recovery from extreme drought, rather than infrequent fire (Burkill, 1985).

We found no evidence of significant spatial bias in our dataset, although temporal bias cannot be excluded. If sampling of a species is temporarily constrained (such as a resprouter that initially dominates, but after successive frequent fire is outcompeted by a seeder), it may falsely be recorded as absent. Changes in fire frequency can alter the composition and abundance of resprouting grass species (Forrestel *et al*., 2014) but how the abundance of grass seeders/resprouters relates to fire frequency remains unknown. In a study of Mediterranean shrubland communities (which include several grasses), the relative abundance of resprouters (verses seeders) increases with fire frequency, whilst the actual abundance of both resprouters and seeders decreases (Vilà‐Cabrera *et al*., 2008).

We found that the ability to resprout post‐fire was not significantly associated with either C_3_ or C_4_ photosynthetic pathways, matching previous work using a smaller sample (Pausas & Paula, 2020). As in that work, we found that bud position was indeed related to resprouting ability, with a significantly higher proportion of resprouting species having buds belowground in the form of rhizomes as predicted. Resprouting from the crown was the most common strategy overall in fire‐prone grasses, suggesting that the tightly packed leaf bases of caespitose grasses effectively protect buds from intense heat (or that buds may be positioned below the soil surface, as in some Andropogoneae species) and stolons are rare, possibly because of the high vulnerability of their buds to fire (above, at, or just below the soil surface). As expected, life history was strongly related to resprouting ability, but interestingly there are exceptions with seeder perennials and disturbance‐tolerant annuals. This latter group was a mixture of C_3_ and C_4_ species, and had crown buds (where we had data). The ecological and environmental causes of these ‘exceptions’ could be an interesting line of study.

Leaf traits relating to N content, but not SLA, were significantly associated with persistence strategy, with resprouters having lower leaf N contents and higher C : N ratios. Due to its low temperature of volatilisation in comparison with other macronutrients (Neary *et al*., 1999), N is selectively lost during fires resulting in typically N‐poor soils (Vitousek & Howarth, 1991; Pellegrini *et al*., 2015). As resprouters may experience fires and low‐N conditions multiple times during their lives, high N‐use efficiency may be under selective pressure, resulting in their lower leaf N contents in comparison with seeders (as has also been found Mediterranean‐climate woody species; Paula & Pausas, 2006; but see Vivian & Cary, 2012). However, these differences could also reflect differences in resource uptake, such that seeders and resprouters segregate along the leaf economics spectrum, with seeders adopting a more resource acquisitive strategy (associated with rapid growth, high photosynthetic rates, short leaf‐life spans and low investment in leaf structure; Wright *et al*., 2004) than resprouters. In addition, differences in drought regimes experienced by seeder and resprouter species could explain this variation in leaf N. Species growing in drier sites, like grass seeders, often show higher leaf N content (Reich *et al*., 1999; Wright *et al*., 2001, 2005), possibly because greater investment in N‐rich photosynthetic machinery may permit higher photosynthetic rates for a given stomatal conductance (Wright *et al*., 2001). The high C : N ratio of grass resprouters enhances their flammability, by reducing decomposition and allowing an accumulation of biomass (Simpson *et al*., 2016). The growth and survival of these species may be enhanced by high flammability, which aids the removal of standing dead and woody biomass (Everson *et al*., 1988; Zimmerman *et al*., 2010).

Fire and drought regimes are changing worldwide. Climate change and human activity have greatly altered global fire, and significantly led to the suppression of fire in grassy environments (Andela *et al*., 2017). Increasing temperatures and vapour pressure deficit means that many areas currently affected by drought will become more arid, and rainfall variability will increase (IPCC, 2014). How these multiple, changing factors will combine to impact grass species distributions is unclear (Settele *et al*., 2014), although our results suggest grass species responses will depend upon their persistence strategy.

## Author contributions

KJS, GHT, CERL, SA and CPO designed the study. KJS, ECJ, EJF and SA generated the data. KJS, GHT and CPO analysed and interpreted the data. KJS wrote the paper with the help of all the authors.

## Supporting information


**Fig. S1** Proportion of grass species occurrence records that fell within burnt pixels against species frequency.
**Fig. S2** Occurrence data for 734 fire‐prone grasses.
**Fig. S3** Principal components analysis biplot for leaf economic spectrum traits.
**Fig. S4** The relationships between fire characteristics and drought.
**Table S1** Number of occurrence records and species represented remaining after each cleaning step.
**Table S2** Sample sizes for analyses representing the overlap of species data for each named trait with data on resprouting ability.
Please note: Wiley Blackwell are not responsible for the content or functionality of any Supporting Information supplied by the authors. Any queries (other than missing material) should be directed to the *New Phytologist* Central Office.

## Data Availability

Plant trait data: Species‐level values for persistence strategy, photosynthetic pathway, bud position, life history and leaf trait PCA scores will be made available in Dryad entry doi: 10.5061/dryad.3bk3j9khn. Fire and drought characteristic data: Species‐level values for fire frequency and intensity, and FDI will be made available in Dryad entry doi: 10.5061/dryad.3bk3j9khn. Phylogeny: The maximum clade credibility tree of the study species will be made available in Dryad entry doi: 10.5061/dryad.3bk3j9khn R code: An example R code to extract and clean occurrence data, extract fire and drought characteristic data, and carry out the phylogenetic logistic regression will be made available in Dryad entry doi: 10.5061/dryad.3bk3j9khn.
