# Supplementary material for: Resprouting grasses are associated with less frequent fire than seeders
Source: New Phytol. 2020 Dec 6;230(2):832–44. doi: 10.1111/nph.17069 (PMC8048952; doi:10.1111/nph.17069)
Supplement: Supplementary file 1 — Fig. S1 Proportion of grass species occurrence records that fell within burnt pixels against species frequency. Fig. S2 Occurrence data for 734 fire‐prone grasses. Fig. S3 Principal components analysis biplot for leaf economic spectrum traits. Fig. S4 The relationships between fire characteristics and drought. Table S1 Number of occurrence records and species represented remaining after each cleaning step. Table S2 Sample sizes for analyses representing the overlap of species data for each named trait with data on resprouting ability. Please note: Wiley Blackwell are not responsible for the content or functionality of any Supporting Information supplied by the authors. Any queries (other than missing material) should be directed to the New Phytologist Central Office. [file NPH-230-832-s001.pdf]

*New Phytologist* **Supporting Information**

Article title: Resprouting grasses are associated with less frequent fire than seeders

Authors: Kimberley J. Simpson, Emma C. Jardine, Sally Archibald, Elisabeth J. Forrestel, Caroline E. R. Lehmann, Gavin H. Thomas & Colin P. Osborne

Article acceptance date: 30 October 2020

The following Supporting Information is available for this article:

**Fig. S1** Proportion of grass species occurrence records that fell within burned pixels against species frequency

**Fig. S2.** Occurrence data for 734 fire-prone grasses.

**Fig. S3.** Principal components analysis biplot for leaf economic spectrum traits.

**Fig. S4**. The relationships between fire characteristics and drought.

**Table S1**. Occurrence records remaining after each cleaning step.

**Table S2.** Sample sizes for analyses.

**Figure S1. Proportion of grass species occurrence records that fell within pixels that burnt (relative to the total number of records) against species frequency**. The estimated breakpoint (0.26), shown by the dashed line (and 95% confidence intervals in grey), was used to define whether species were fire-prone or not (i.e. a species was considered 'fire-prone' if ≥ 26% of its occurrence records fell in an area that burnt at least once during the MODIS global monthly burnt area satellite dataset).

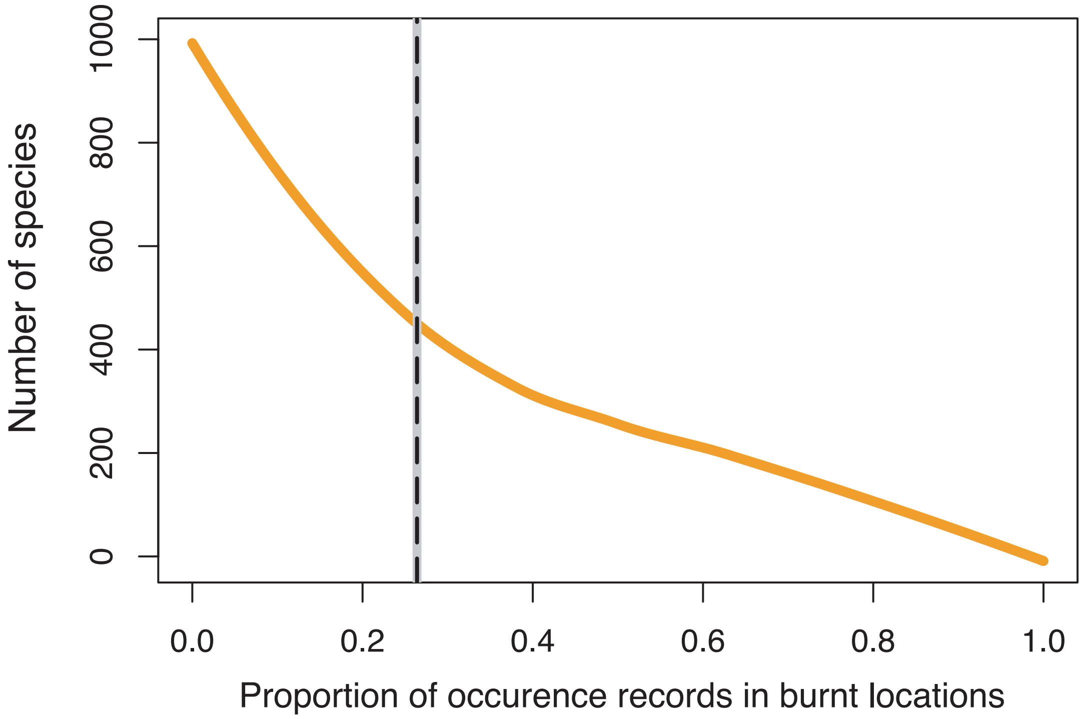

**Figure S2. Occurrence data for 734 fire-prone grasses.** Black dots are point occurrences for individual records that were downloaded from the Global Biodiversity Facility (GBIF).

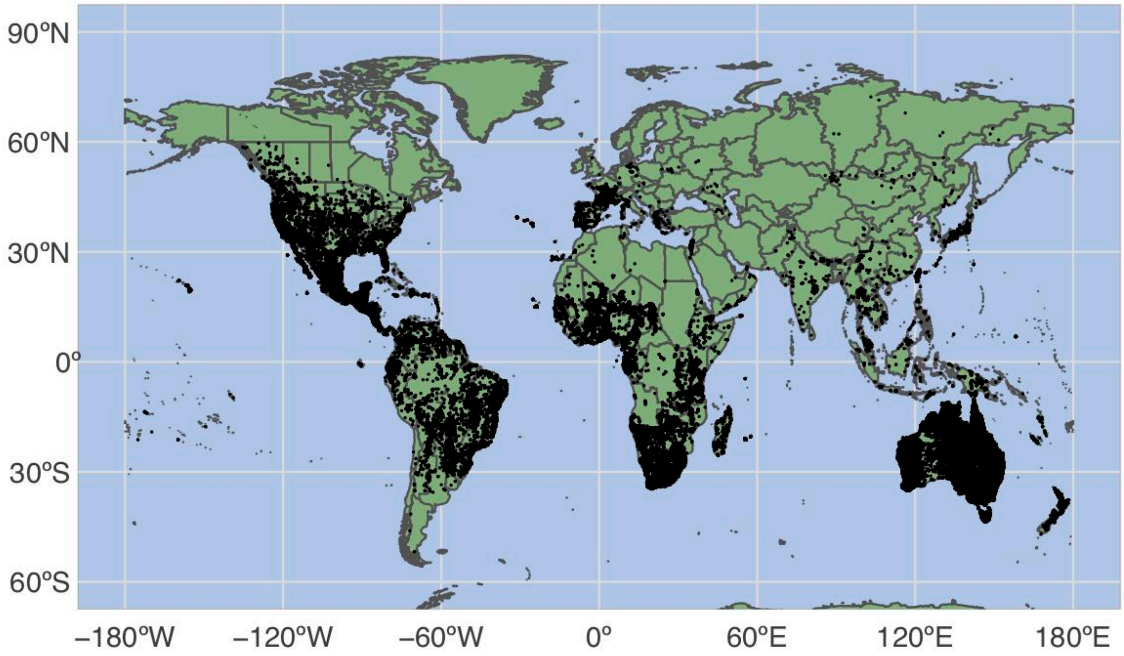

**Figure S3. Principal components analysis biplot for leaf economic spectrum traits.** All parameters were log-transformed to improve normality. SLA = specific leaf area; C:N = leaf carbon to nitrogen ratio; Leaf N = leaf nitrogen content.

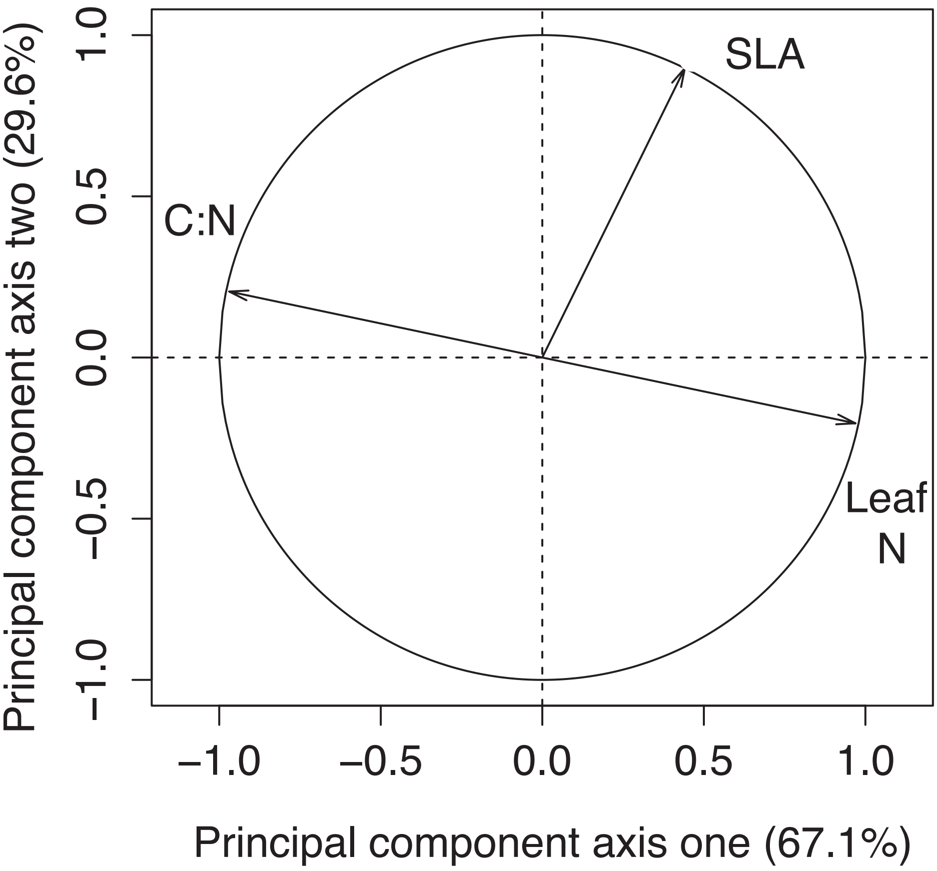

**Figure S4. The relationships between fire characteristics and drought across fire-prone grass species.** Fire frequency (measured as median fire return interval) was significantly related to fire intensity (95th percentile fire radiative power, a), and drought (characterized by Foley's drought index (Foley, 1957) with more negative values representing more extreme drought; b). Fire intensity was significantly related to drought, with higher fire intensities associated with places that experience more extreme drought (c). Each point represents one grass species. Relationships were extracted from phylogenetic generalised least squares models.

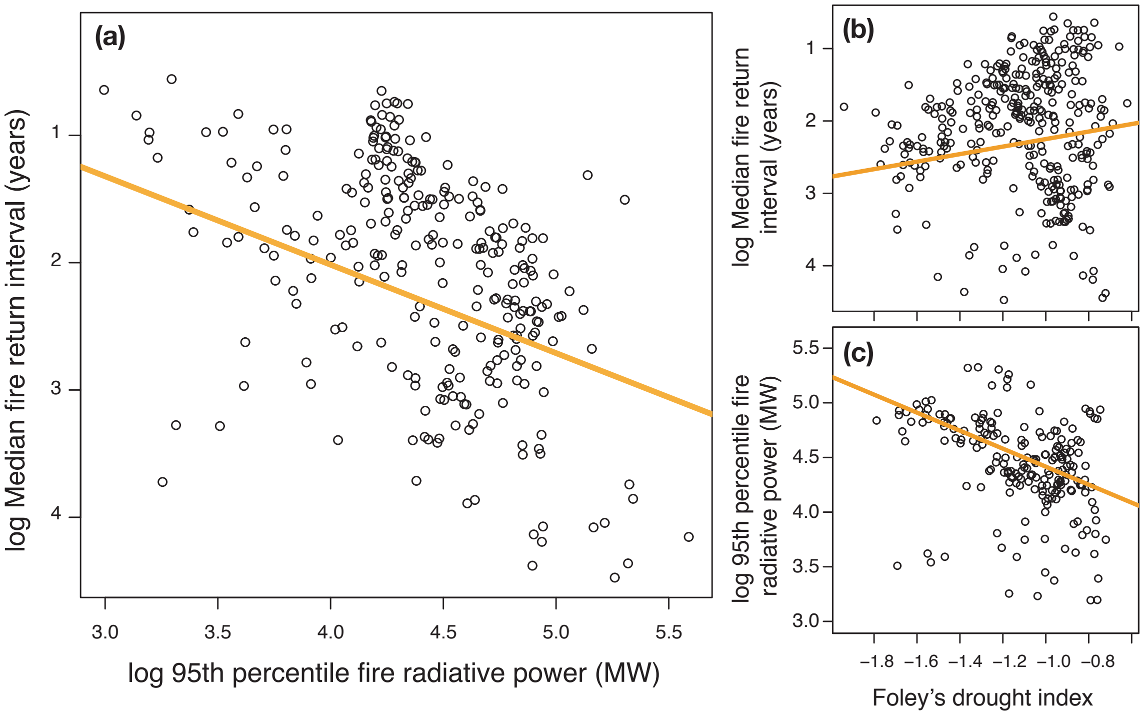

**Table S1.** The number of occurrence records and species represented remaining after each cleaning step.

| Cleaning step | Number of records remaining after step (millions) | Number of species remaining after step |
|---|---|---|
| Original data download | 18.6 | 10051 |
| Cleaning using ‘CoordinateCleaner’ R package* | 17.5 | 9884 |
| Removing cultivated species | 17.2 | 9846 |
| Removing records where latitude/longitude aren’t accurate to 3 decimal places | 12.5 | 9025 |
| Removing records found outside a species native range | 10.9 | 7179 |
| Removing recods from highly transformed landscapes (outside of World Protected Areas and Human Influence Index score > 30) | 7.5 | 5540 |
| Removing records from before 1980 | 4.8 | 2584 |
| Removing records of species with <50 occurrences | 2.8 | 1703 |

* using tests: ‘centroids’, ‘equal’, ‘gbif’, ‘institutions’, ‘seas’ and ‘zeros’; Zizka et al. (2019)

**Table S2.** Sample sizes of datasets for analyses.

| Trait | Number of species |
|---|---|
| Fire frequency § | **332** |
| Fire intensity* | **550** |
| Bud position† | **561** |
| Leaf economic spectrum traits† | **114** |
| Life history† | **752** |
| Photosynthetic pathway† | **597** |

§ representing the overlap of species fire frequency data with data on drought and grass persistence strategy. Species for which fire return intervals couldn't be determined or were not reliable based on the length of the MODIS dataset (>100yrs) were excluded from the analysis.

* representing the overlap of species fire intensity data with data on drought and grass persistence strategy

† representing the overlap of species data for each named trait with data on resprouting ability.

References

**Foley JC. 1957.** Droughts in Australia. Review of Records from Earliest Years of Settlement to 1955. Bulletin No. 47. Bureau of Meteorology, Commonwealth of Australia, Melbourne, Australia.

**Zizka A, Silvestro D, Andermann T, Azevedo J, Ritter CD, Edler D, Farooq H, Herdean A, Ariza M,Scharn R, et al. 2019.** CoordinateCleaner: Standardized cleaning of occurrence records from biological collection databases. *Methods in Ecology and Evolution* **10**: 744– 751.
